# Pan-Src kinase inhibitor treatment attenuates diabetic kidney injury via inhibition of Fyn kinase-mediated endoplasmic reticulum stress

**DOI:** 10.1038/s12276-022-00810-3

**Published:** 2022-08-02

**Authors:** Debra Dorotea, Songling Jiang, Eun Seon Pak, Jung Beom Son, Hwan Geun Choi, Sung-Min Ahn, Hunjoo Ha

**Affiliations:** 1 Graduate School of Pharmaceutical Sciences, College of Pharmacy, Ewha Womans University, Seoul, Republic of Korea; 2 New Drug Development Center, Daegu-Gyeongbuk Medical Innovation Foundation, Daegu, Republic of Korea; 3 ImmunoForge, Seoul, Republic of Korea

**Keywords:** Mechanisms of disease, Chronic inflammation, Biologics, Chronic kidney disease

## Abstract

Src family kinases (SFKs) have been implicated in the pathogenesis of kidney fibrosis. However, the specific mechanism by which SFKs contribute to the progression of diabetic kidney disease (DKD) remains unclear. Our preliminary transcriptome analysis suggested that SFK expression was increased in diabetic kidneys and that the expression of Fyn (a member of the SFKs), along with genes related to unfolded protein responses from the endoplasmic reticulum (ER) stress signaling pathway, was upregulated in the tubules of human diabetic kidneys. Thus, we examined whether SFK-induced ER stress is associated with DKD progression. Mouse proximal tubular (mProx24) cells were transfected with *Fyn* or *Lyn* siRNA and exposed to high glucose and palmitate (HG-Pal). Streptozotocin-induced diabetic rats were treated with KF-1607, a novel pan-Src kinase inhibitor (SKI) with low toxicity. The effect of KF-1607 was compared to that of losartan, a standard treatment for patients with DKD. Among the SFK family members, the Fyn and Lyn kinases were upregulated under diabetic stress. HG-Pal induced p70S6 kinase and JNK/CHOP signaling and promoted tubular injury. *Fyn* knockdown but not *Lyn* knockdown inhibited this detrimental signaling pathway. In addition, diabetic rats treated with KF-1607 showed improved kidney function and decreased ER stress, inflammation, and fibrosis compared with those treated with losartan. Collectively, these findings indicate that Fyn kinase is a specific member of the SFKs implicated in ER stress activation leading to proximal tubular injury in the diabetic milieu and that pan-SKI treatment attenuates kidney injury in diabetic rats. These data highlight Fyn kinase as a viable target for the development of therapeutic agents for DKD.

## Diabetes: A new approach to preventing kidney damage

Insights into a signaling pathway that promotes diabetic kidney disease could lead to new therapies that protect against this major cause of kidney failure. Past studies have suggested that the various Src family kinase (SFK) signaling proteins play a part in the cell death and scar tissue formation associated with diabetic kidney disease. Hunjoo Ha of Ewha Womans University, Seoul, South Korea, and colleagues have now focused on one particular SFK, Fyn, as a direct driver of the kidney damage seen in mouse models of diabetes. Genetic interventions that selectively inhibit Fyn suppressed this damage, as did treatment with an oral drug that broadly inactivates SFKs. This experimental drug proved as effective as controlling inflammation and oxidative damage in the kidney as an already clinically approved treatment, confirming the significance of SFK signaling in this condition.

## Introduction

Diabetic kidney disease (DKD) has been recognized as a major cause of chronic kidney disease (CKD) and end-stage kidney disease worldwide^1^. The mainstay treatments for DKD include glycemic control, blood pressure control, and blockade of the renin–angiotensin-aldosterone system (RAAS). However, the staggering prevalence of DKD implies that this strategy may not fully target the underlying pathogenic mechanism of DKD^2^. Sodium-glucose cotransporter 2 (SGLT2) inhibitors have been recently added as a treatment option for DKD^3^. However, a better understanding of the pathogenesis of DKD remains important to develop novel therapeutic agents.

Accumulating evidence suggests that Src family kinases (SFKs) play an important role in the development of CKD. The SFKs, a family of nonreceptor tyrosine kinases, comprise the ubiquitously expressed c-Src, Fyn, Yes, and Yrk kinases and the Lyn, Fgr, Hck, Blk, and Lck kinases, which are primarily expressed in hematopoietic cells^4^. Src kinase activation by various injurious stimuli has been widely demonstrated to stimulate multiple fibrotic signaling pathways in the kidney^5–7^. The role of other SFKs has also been reported in several kidney diseases. Mice with Fyn gene ablation showed attenuation of tubulointerstitial fibrosis^8^, while Hck expression was elevated in mice with DKD, IgA nephropathy, and lupus nephritis^9^. Pan-Src kinase inhibitors (SKIs), including clinically used drugs (e.g., dasatinib and nintedanib), indeed showed protective effects in mice with CKD^7,9–11^ and DKD^6,12^.

Most studies in the kidney have focused on Src-induced activation of fibrotic signaling pathways, including TGF-β1/Smad3, EGFR, and STAT3^7^. However, Src and Fyn kinases have also been demonstrated to induce endoplasmic reticulum (ER) stress^13–15^, which is important to maintain cellular survival in early disease but can be cytotoxic under chronic disease conditions^16^. Furthermore, Fyn was identified among the putative genes enriched in the proximal tubules of the human kidney that are associated with endolysosomal pathway dysfunction in CKD development^17^. Since aggravated ER stress is a crucial mechanism mediating DKD progression^18^, the present study investigated whether SFK-mediated ER stress activation contributes to the progression of DKD.

Protective effects of SKIs against DKD have been shown mainly in animal studies using an experimental pan-SKI that is administered intravenously, thereby limiting the feasibility of daily or long-term administration. Moreover, these studies did not compare the efficacy of SKIs with that of clinically used drugs for DKD^6,12^. Our previous study suggested that KF-1607, a newly synthesized oral pan-SKI, prevented the progression of tubulointerstitial fibrosis in mice with obstructive nephropathy^10^. To promote SFKs as therapeutic targets in DKD, the present study examined the protective effects of KF-1607 in comparison with those of losartan.

## Materials and methods

### Chemicals and reagents

All chemicals used in the present study were obtained from Sigma-Aldrich (St. Louis, MO, USA), unless otherwise stated.

### Cell culture

Immortalized mouse proximal tubular (mProx24) cells were supplied by Dr. Takeshi Sugaya (St. Marianna University School of Medicine, Kanagawa, Japan), and NRK52E rat kidney epithelial cells were purchased from the American Type Culture Collection (Manassas, VA, USA). Both cell lines were maintained in DMEM (Gibco, Grand Island, NY, USA) supplemented with 10,000 U/ml penicillin-streptomycin (Gibco) and fetal bovine serum (FBS) (Gibco; 10 and 5% for mProx24 and NRK52E cells, respectively) at 37 °C in a humidified atmosphere of 5% CO_2_. For each experiment, subconfluent mProx24 cells were starved by incubation in DMEM containing 1% FBS for 24 h followed by incubation with or without 55.5 mM (high) glucose and 150 mM palmitate bound to 10% bovine serum albumin (BSA) (HG-Pal) for 10 h. Subconfluent NRK52E cells were starved by incubation in DMEM containing 0.1% FBS for 24 h followed by pretreatment with either KF-1607 or PP2 (Calbiochem, San Diego, CA, USA) for 3 h and incubation with or without HG-Pal for 18 h. The concentration of HG-Pal used in the present study was determined based on our preliminary study.

### siRNA transfection

mProx24 cells were grown to a confluence of greater than 50% and transfected with 150 nM *Fyn* and *Lyn* siRNA duplexes (Bioneer, Daejeon, Republic of Korea) using Lipofectamine RNAiMAX Reagent (Invitrogen, Carlsbad, CA, USA) for 18 h. The sequences were as follows: *Fyn* siRNA, 5′-GUGAACUCCUCUCAC-3′; *Lyn* SiRNA, 5′-CUACUAAUCCAGCCUGUUA-3′. Scrambled siRNA (Bioneer) was used as the control. Transfected cells were then treated with HG-Pal for 10 h.

### Animal experiments

All animal experiments were approved by the Institutional Animal Care and Use Committee of Ewha Womans University (IACUC-14-109). Six- to seven-week-old male Sprague–Dawley (SD) rats (Japan SLC Inc., Hamamatsu, Japan) were housed in a room maintained at 22 ± 2 °C on a 12 h dark/12 h light cycle. Type 1 diabetes mellitus was induced by a single intraperitoneal injection of 60 mg/kg streptozotocin (STZ). Age-matched control rats were injected with an equal volume of sodium citrate buffer (100 mM sodium citrate, 100 mM citric acid, pH 4.5).

A novel pan-SKI, KF-1607, was developed and synthesized by the New Drug Development Center, Daegu-Gyeongbuk Medical Innovation Foundation (Daegu, Republic of Korea) and ImmunoForge (Seoul, Republic of Korea). KF-1607 is a small molecule inhibitor of SFKs, including Blk, c-Src, Fyn, Hck, Lck, Lyn, and Yes, with IC50 values of 17.3, 0.788, 1.31, 2.42, 1.02, <0.508, and <0.508 nM, respectively. The synthesis and characteristics of the compounds are described in our previous publication^10^. To examine the protective effect of pan-SKI treatment, KF-1607 (30 mg/kg) was administered to diabetic rats for 8 weeks by oral gavage. For comparison, diabetic rats were treated with losartan (1 mg/kg, per oral), an angiotensin receptor blocker (ARB). The dose of losartan was determined based on our previous study^19^. Nondiabetic rats were administered an equal volume of the vehicle used for KF-1607 preparation (DMSO:Tween 70:DW 10:5:85). All rats were euthanized after 8 weeks of drug treatment.

### Measurement of blood parameters

Random blood glucose testing was conducted using a glucometer (OneTouch Ultra, Johnson & Johnson Co., CA, USA) every other week during the experiment. Blood samples were collected before sacrifice. Hemoglobin A1c (HbA1c) levels were measured using DCA2000 HbA1c reagent kits (Siemens Healthcare Diagnostics, Inc., Tarrytown, NY, USA). Blood samples were centrifuged at 900 × *g* for 15 min at 4 °C to collect plasma. Serum creatinine and free fatty acid (FFA) levels were measured using ELISA kits obtained from Arbor Assays (Ann Arbor, MI, USA) and BioAssay Systems (Hayward, CA, USA), respectively.

### Measurement of urine parameters

Before the day of sacrifice, 24-h urine samples were collected from the rats housed in metabolic cages, and the collected samples were centrifuged at 900 × *g* for 10 min. Urinary excretion of albumin and kidney injury molecule 1 (KIM-1) were measured using ELISA kits provided by ALPCO (Westlake, OH, USA) and R&D Systems (Minneapolis, MN, USA), respectively.

### Measurement of lipid peroxide (LPO) levels

Plasma and urinary LPO levels were measured as thiobarbituric acid-reactive substances^20^. Briefly, plasma or urine aliquots were mixed with 8% SDS and a mixture of 0.8% 2-thiobarbituric acid and 20% acetic acid. These mixtures were incubated at 95 °C for 60 min. After cooling, the samples were centrifuged at 1600 × *g* for 5 min to precipitate interfering particulate materials. The LPO level was measured using a spectrofluorometer (Thermo Fisher Scientific, Waltham, MA, USA). The LPO concentration in the kidney was measured using an LPO assay kit (Cayman Chemical, Ann Arbor, MI, USA) according to the manufacturer’s instructions.

### Histological and immunohistochemical staining

Kidneys were fixed with 2% paraformaldehyde-lysine-periodate (pH 7.4), dehydrated, and embedded in paraffin. Kidney tissue sections (5 μm) were stained with Masson’s trichrome and picrosirius red (Abcam, Waltham, MA, USA) to visualize the collagen matrix. Kidney tissue sections were also stained with periodic acid-Schiff reagent to quantitatively analyze the tuft area, glomerular volume, and fractional mesangial area (FMA) in the stained glomeruli, as previously described^19^. In addition, kidney tissue sections were immunostained with primary antibodies, as mentioned in Supplementary Table 1. Tissue sections were deparaffinized and incubated sequentially with 3% hydrogen peroxide and 5% normal horse serum, avidin/biotin blocking solution (Vector Laboratories, Burlingame, CA, USA), a primary antibody (overnight at 4 °C), and a specific secondary antibody (Vector Laboratories). Bound antibodies were visualized with 3,3-diaminobenzidine (DAB; Dako, Glostrup, Denmark). Images were acquired using a Zeiss microscope equipped with an AxioCam HRC digital camera and AxioCam software (Zeiss, Thornwood, NY, USA), and the indicated parameters were quantified with Image-Pro Plus 4.5 software (Cybernetics, Silver Spring, MD, USA).

### Immunofluorescence staining

After deparaffinization and rehydration, kidney tissue sections were incubated with retrieval solutions and heated in a microwave for antigen retrieval. Nonspecific binding was blocked with serum-free blocking solution for 30 min at room temperature. After overnight incubation with primary antibodies at 4 °C, the tissue sections were labeled with Alexa Fluor 488- and Alexa Fluor 568-conjugated secondary antibodies (1:1000, Invitrogen) in the dark for 1 h at room temperature. Nuclei were detected with 4,6-diamidino-2-phenylindole (DAPI; 1:1000, Thermo Fisher Scientific).

For immunofluorescence staining, cells grown on chamber slides were fixed with 4% paraformaldehyde for 20 min at room temperature, permeabilized using 0.5% Triton X-100 for 10 min, and blocked with 3% BSA for 30 min at room temperature. After overnight incubation with primary antibodies in 3% BSA at 4 °C, the cells were rinsed with PBS and labeled with fluorescent secondary antibodies in the dark for 1 h at room temperature. Cells were washed twice with PBS and incubated with DAPI in PBS at room temperature for 5 min. Images were acquired using a Zeiss Apotome.2 (Carl Zeiss). Detailed information regarding the primary antibodies used for immunofluorescence staining is provided in Supplementary Table 1.

### Immunoblot analysis

Protein concentrations in kidney tissue homogenates and cell lysates were measured using the Bradford method (BioRad Laboratories, Hercules, CA, USA). Proteins were separated by electrophoresis and subsequently transferred onto PVDF membranes (GE Healthcare BioSciences Co., Piscataway, NJ, USA). The membranes were blocked with 5% skim milk in TBS-Tween 20 buffer for 1 h at room temperature and then incubated with primary antibodies at 4 °C overnight. The membranes were subsequently incubated with a horseradish peroxidase-conjugated secondary antibody for 1 h at room temperature, and the blots were developed with enhanced chemiluminescence detection reagent (GE Healthcare BioSciences Co.). Positive immunoreactive bands were detected using an enhanced chemiluminescence method (LAS-3000, Fujifilm, Tokyo, Japan). Detailed information regarding the primary antibodies used for immunoblotting is provided in Supplementary Table 1.

### Real-time PCR

Total cellular RNA was extracted using TRIzol reagent (Invitrogen). Real-time PCR was performed using an ABI7300 system (Applied Biosystems, Carlsbad, CA, USA) with a 20 μl reaction volume consisting of cDNA transcripts, primer pairs, and SYBR Green PCR Master Mix (Applied Biosystems, Carlsbad, CA, USA). 18S rRNA was used as the internal control, and the primer sequences (Bioneer) used in the present study are listed in Supplementary Table 2.

### Database analysis

Transcriptome data obtained from the Nephroseq database (nephroseq.org) were analyzed to identify gene expression patterns in the selected renal disease^21^. In the “Woroniecka Diabetes” dataset (GSE30122), Affymetrix expression arrays were used to identify differentially regulated transcripts in 22 microdissected human kidney tubule samples, among which ten were collected from patients with DKD^22^. The “Nakagawa Kidney” dataset (GSE66494) includes microarray gene expression profiles of biopsy specimens from both normal healthy kidneys (*n* = 5) and CKD kidneys (*n* = 48)^23^. The “Hodgin Diabetes Mouse” dataset (GSE33744) includes glomerular gene expression profiles in streptozotocin-induced diabetic DBA/2 mice (*n* = 17) and db/db C57BLKS (*n* = 10) mice^24^. The Reactome database (http://www.reactome.org)^25^ was used to analyze the molecular pathways in which gene IDs from the “Woroniecka Diabetes Tubulointerstitial Study” (GSE30122) were strongly enriched. Pathways with a false discovery rate-corrected *p* value of <0.05 are presented.

### hERG safety assay

hERG activity was examined using an IonWorks Barracuda automated patch clamp system (Molecular Devices, San Jose, CA, USA). Cells stably expressing hERG channels were suspended in the extracellular solution (a mixture of 136.9 mM NaCl, 2.7 mM KCl, 8.1 mM Na_2_HPO_4_, 1.5 mM KH_2_PO_4_, 0.9 mM CaCl_2_, and 0.5 mM MgCl_2_ at pH 7.1) and dispensed into a 384-well patch clamp plate. Cell membranes were perforated by incubation with amphotericin B in the intracellular solution (a mixture of 120 mM KCl, 1.7 mM MgCl_2_, 5 mM CaCl_2_, 10 mM HEPES, and 10 mM EGTA at pH 7.0; osmolarity and pH were adjusted with sucrose and KOH, respectively). The activity of hERG in the negative control wells (external buffer with the addition of 0.2% DMSO) was measured. hERG activity was measured with the following recording protocol: starting at a holding potential of –80 mV, a voltage pulse was applied sequentially at –70 mV (0.2 s), +40 mV (1 s), and –50 mV (1 s). The change in the current in the hERG channel was measured in triplicate. Then, the hERG current following the addition of the test compound was measured; and the duration of incubation with the compound was 10 min. Quinidine was used as the positive control in the current measurements. hERG activity was calculated as the electric current post-compound treatment divided by the electric current pre-compound treatment and is expressed as a percentage.

### Statistical analysis

All results are expressed as the means ± standard errors. Analysis of variance followed by Fisher’s post-hoc test was used to assess differences among multiple groups. Differences were considered significant when the *p* value was <0.05.

## Results

### The expression of Fyn and Lyn kinases is elevated in diabetic kidneys

**Fig. 1:**
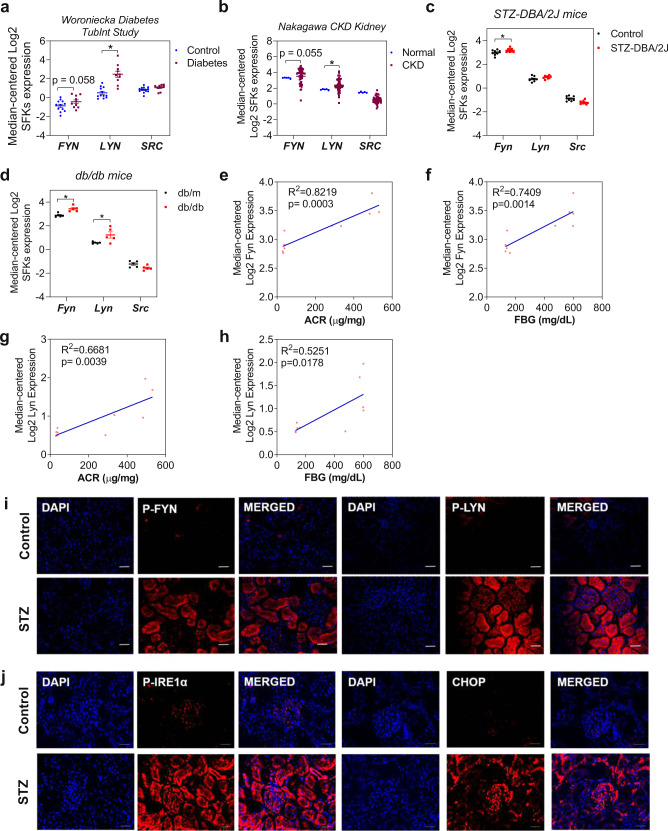
SFKs are important mediators of chronic kidney diseases. Microarray analyses of human and murine kidney biopsies were obtained from the Nephroseq database (nephroseq.org). Comparative transcriptomic data of SFKs were obtained: **a** isolated tubules of diabetic kidneys vs. those of healthy kidneys, **b** biopsy specimens of healthy kidneys vs. biopsy specimens of pathologically confirmed CKD, **c** kidneys of normal control vs. STZ-DBA/2J mice, and **d** kidneys of control db/m vs. db/db mice. Correlation analysis of *Fyn* mRNA expression with the **e** ACR and **f** FBG level in the *Hodgin* dataset with control and db/db mice. Correlation analysis of *Lyn* mRNA expression with the **g** ACR and **h** FBG level in the same dataset. The expression patterns of **i** active, phosphorylated Fyn and Lyn kinases as well as **j** ER stress signaling mediators, represented by p-IRE1 and the CHOP protein, were shown by immunofluorescence staining of kidney sections from rats with diabetes induced for 8 weeks. The white scale bar indicates 50 μm.

As mentioned above, most previous animal studies of CKD demonstrated the increased expression of Src kinase in the kidney without showing the expression of other SFK members. However, our previous study indicated that the expression of several SFKs, i.e., Src, Fyn, and Lyn kinases, was elevated in mice with UUO, a model of progressive CKD^10^. Transcriptome profiling data obtained from the Nephroseq database showed that instead of *SRC*, *FYN* and *LYN* were upregulated in the tubules of diabetic patients with a decreased glomerular filtration rate (Fig. 1a) and in the kidneys of CKD patients with progressive fibrosis (Fig. 1b). Increased expression of *Fyn* and *Lyn* was also found in the diabetic kidneys of STZ-induced mice (Fig. 1c) and db/db mice (Fig. 1d). *Fyn* mRNA expression in the *Hodgin* dataset showed a stronger correlation with kidney function and diabetic state than did *Lyn* mRNA expression. Increased transcript levels of *Fyn* were observed in db/db mice with higher albumin/creatinine ratios (ACRs) and plasma fasting blood glucose (FBG) levels (Fig. 1e–h). However, no significant correlation was shown between the *Src* transcript level and the ACR and FBG in this dataset (Supplementary Fig. 1a, b).

Moreover, it has been reported that Fyn kinase induces ER stress signaling in skeletal muscle^13^. Based on analysis of the “Woroniecka Diabetes Study” dataset, the ER–phagosome pathway, interleukin–interferon signaling, and extracellular matrix (ECM) organization were among the enriched biochemical pathways overexpressed in the diabetic kidney. Coexpression analysis further indicated that the expression levels of *Fyn* and unfolded protein response (UPR)-related genes were elevated in the tubules of human diabetic kidneys (Supplementary Fig. 2a, b). The present study therefore aimed to determine the mechanistic role of SFKs in inducing ER stress and DKD.

To further corroborate the above transcriptomic findings, the expression levels of Fyn and Lyn kinases in the kidneys of diabetic rats were evaluated. Immunostaining of kidney sections from rats with diabetes induced for 8 weeks showed increased signals of phosphorylated Fyn and Lyn kinases in glomeruli and tubules, with phosphorylated Fyn kinase prominently detected in tubules (Fig. 1i). Exacerbated ER stress was also observed in diabetic kidneys, as indicated by the increased signals of phosphorylated IRE-1α and CHOP (Fig. 1j).

### High glucose–palmitate exposure induces Fyn and Lyn kinase phosphorylation along with ER stress signaling activation in mouse proximal tubular cells

**Fig. 2:**
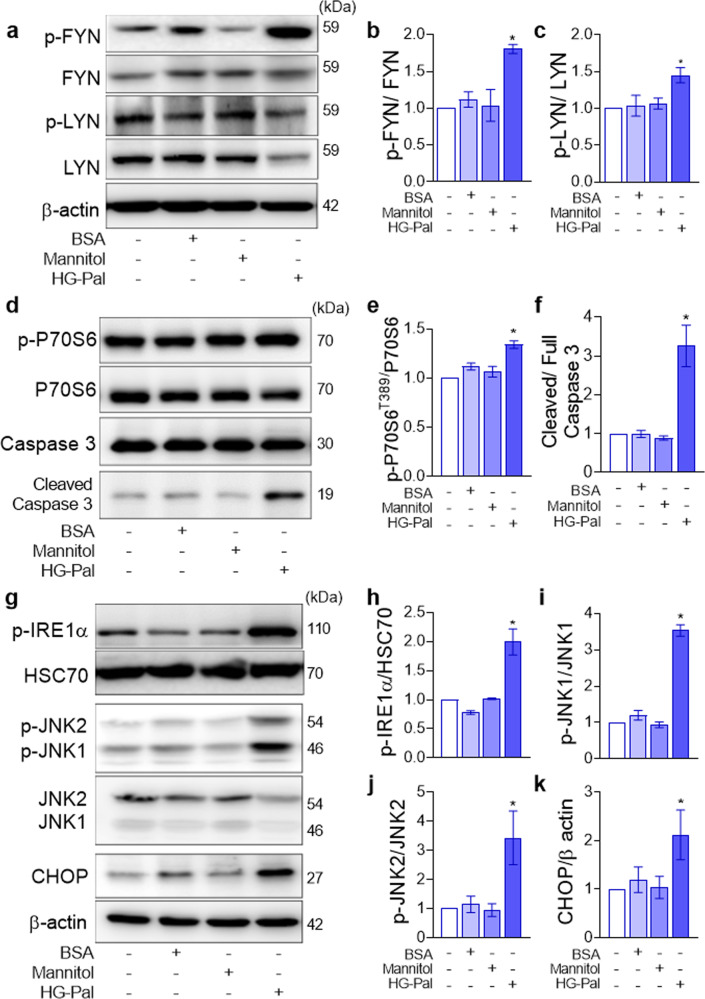
Fyn and Lyn kinase expression, along with ER stress signaling, is enhanced in mouse proximal tubular cells exposed to high glucose and palmitate. mProx24 cells were treated with HG-Pal for 10 h, and cell lysates were subjected to immunoblot analysis. **a** The levels of activated, phosphorylated Fyn and Lyn kinases were measured by immunoblotting. Activation of mTORC1 and apoptosis was indicated by increases in the levels of **d** phosphorylated p70S6K and cleaved caspase 3. **g** Upregulation of the UPR signaling pathways during ER stress was indicated by the increases in the levels of phosphorylated IRE1α, phosphorylated JNK1/2^T183, Y185^, and CHOP. **b**, **c**, **e**, **f**, **h**–**k** The expression levels of the indicated proteins were quantified by densitometry. The data are presented as the mean ± SE of four experiments. *p < 0.05 vs. control.

The combination of high glucose and palmitate (HG-Pal) mimics the diabetic milieu and is associated with renal cell apoptosis and mTORC1 activation, which are linked to ER stress activation^26,27^. To determine the possible mechanism by which SFKs mediate ER stress-induced diabetic kidney injury, an in vitro study with mouse proximal tubular cells under hyperglycemic and lipotoxic conditions was performed. mProx24 cells exposed to HG-Pal showed significant increases in Fyn and Lyn kinase phosphorylation (Fig. 2a–c). HG-Pal-treated mProx24 cells exhibited increased mTORC1 activation, as indicated by the increase in the phosphorylation of p70S6 kinase (p70S6K), a major downstream target of mTORC1 (Fig. 2d, e). The increased cleaved caspase 3 protein expression further suggested tubular apoptosis in HG-Pal-treated mProx24 cells (Fig. 2d, f). Accordingly, HG-Pal-treated mProx24 cells exhibited enhanced ER stress signaling, as shown by the significant increases in the levels of phosphorylated IRE1α, phosphorylated JNKs, and CHOP, a transcription factor responsible for ER stress-related apoptosis (Fig. 2g–k). Other independent downstream signaling pathways of the UPR were examined, and the protein expression level of phosphorylated PERK but not ATF6α was increased under our experimental conditions (Supplementary Fig. 3a–c).

### Knockdown of Fyn attenuates ER stress and protects against tubular injury in mProx24 cells exposed to HG–palmitate

**Fig. 3:**
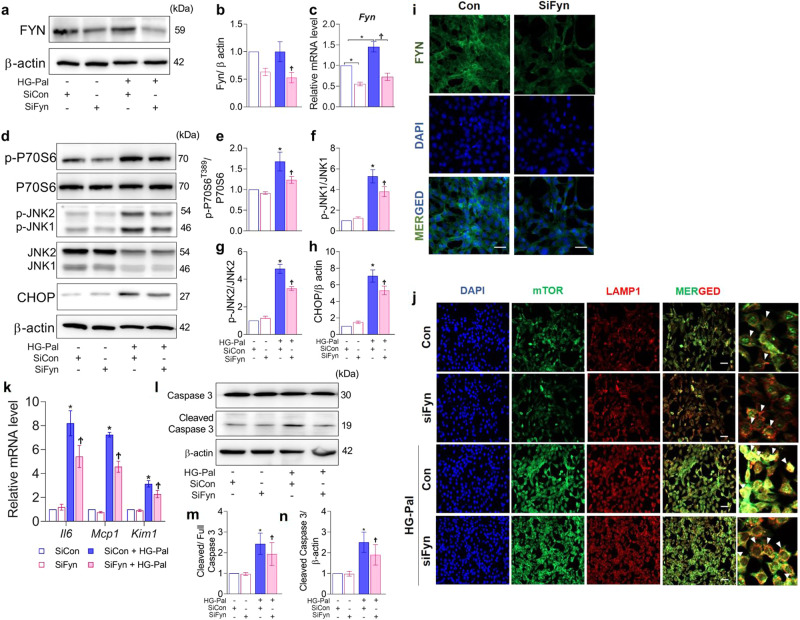
Knockdown of *Fyn* attenuates ER stress and protects against tubular injury in mProx24 cells under diabetic stress. mProx24 cells were transfected with scrambled siRNA or *Fyn* siRNA and stimulated with or without HG-Pal. The knockdown efficiency was verified by measuring the **a** protein expression level and **c** transcript level of *Fyn*. **d** The expression levels of proteins related to mTORC1 activation and ER stress signaling were examined in cells with *Fyn* knockdown and HG-Pal exposure. **i** Immunostaining of cells transfected with scrambled siRNA and Fyn siRNA. **j** Coimmunostaining of mTOR and LAMP-1, a lysosomal membrane marker, in cells with *Fyn* knockdown with/without HG-Pal stimulation. The yellow pixels indicate colocalization in the merged images. **k** The decreases in the transcript levels of *Il6*, *Mcp1*, and *Kim1* as well as **l** the protein level of cleaved caspase 3 suggested the protective effects of *Fyn* knockdown in tubules. **b**, **e**–**h**, **m**, **n** The expression levels of the indicated proteins were quantified by densitometry. The data are presented as the mean ± SE of four experiments. *p < 0.05 vs. siCon, ^†^*p* < 0.05 vs. HG-Pal and siCon.

Genetic ablation of *Fyn* was utilized to investigate the contribution of Fyn kinase to tubular injury under diabetic stress conditions. *Fyn*-specific siRNA transfection significantly decreased the transcript level and protein levels of Fyn kinase in mProx24 cells (Fig. 3a–c) without affecting the expression levels of Lyn kinase (Supplementary Fig. 3d). *Fyn* knockdown led to inhibition of p70S6K, a major downstream target of mTORC1, and decreases in the levels of phosphorylated JNK and CHOP in the ER stress signaling axis (Fig. 3d–h). However, HG-Pal-induced PERK phosphorylation was not suppressed by *Fyn* knockdown under our experimental conditions (Supplementary Fig. 3f, g). It has been reported that Src kinase can induce mTORC1 recruitment and activation at the lysosomal surface^28^. Here, we showed that HG-Pal induced mTOR translocation to the lysosomal membrane (marked by LAMP-1) and that this effect was inhibited by Fyn knockdown (Fig. 3i, j).

Furthermore, HG-Pal treatment significantly increased tubular inflammation and injury, as indicated by the elevated mRNA levels of *Il6*, *Mcp1*, and *Kim1*, which were downregulated by *Fyn*-specific siRNA transfection (Fig. 3k). *Fyn* knockdown in HG-Pal-treated cells also inhibited cellular apoptosis, as suggested by the decrease in the cleaved caspase 3 protein level (Fig. 3l–n).

Since Lyn kinase was also stimulated under HG-Pal treatment, we investigated whether *Lyn* siRNA transfection could lead to suppression of the aforementioned signaling pathway. *Lyn* siRNA transfection significantly downregulated Lyn kinase protein expression (Supplementary Fig. 4a, b) without affecting Fyn kinase expression (Supplementary Fig. 3e). Downregulation of *Lyn* was not associated with protection against injury. Activation of p70S6K and the ER stress signaling axis, i.e., phosphorylation of JNK and CHOP, was not affected by *Lyn* knockdown. Consistent with these results, the cleaved caspase 3 level was not decreased (Supplementary Fig. 4c–h).

### Pan-Src kinase inhibition improves kidney function and attenuates kidney histopathological changes in STZ-induced diabetic rats

Since there is no preceding study of chronic oral pan-SKI therapy in DKD, the efficacy of pan-SKI treatment in preventing the progression of diabetic kidney injury remains important to be examined. We determined the renoprotective effects of KF-1607, a new small molecule inhibitor that has been demonstrated to significantly prevent the progression of tubulointerstitial fibrosis in an obstructive nephropathy model^10^. The hERG potassium channel automated patch clamp assay showed that the IC50 of KF-1607 was 1.1 mM, which was considered to indicate a relatively low potential for cardiotoxicity (IC50 > 10 μM) (Supplementary Fig. 5).

**Fig. 4:**
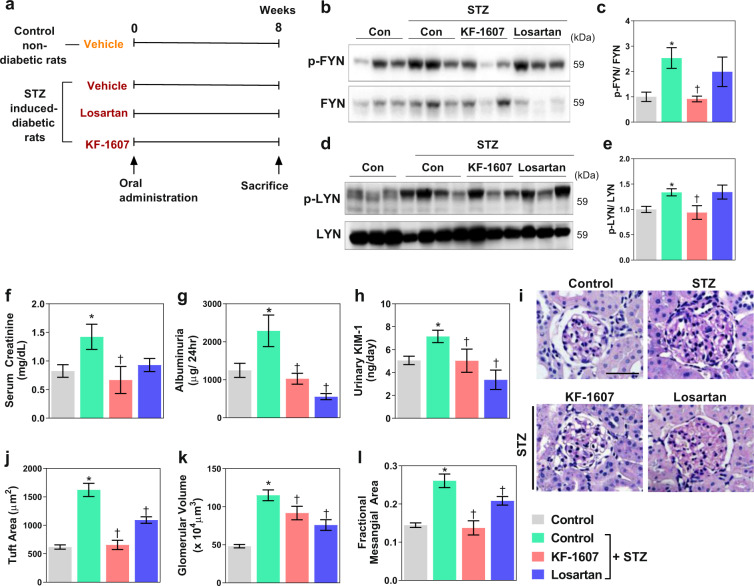
Src kinase inhibition improves kidney function and prevents kidney morphological changes. **a** The scheme of the experimental design. Diabetic rats were treated with either KF-1607 or losartan by oral gavage for 8 weeks. **b**, **d** The expression of Fyn and Lyn kinases was determined in kidney lysates from experimental animals. **c**, **e** The expression levels of the indicated proteins were quantified by densitometry. Following 8 weeks of drug treatment, urine and blood were collected for analysis of **f** serum creatinine, **g** albuminuria, and **h** urinary KIM-1. **i** Kidney sections were stained with PAS to observe pathological changes. Quantitative analysis of the **j** tuft area, **k** glomerular volume, and **l** fractional mesangial area (20–30 glomeruli were counted per mouse). The black scale bar indicates 50 μm. The data are presented as the mean ± SE of five rats. *p < 0.05 vs. control rats. ^†^*p* < 0.05 vs. control-STZ rats.

Diabetic rats received 8 weeks of treatment with either KF-1607 or losartan, a major drug used for preventing the progression of DKD in the clinical setting (Fig. 4a). The protein levels of phosphorylated Fyn and Lyn kinases were significantly increased in the kidney homogenate from diabetic rats and were decreased following KF-1607 treatment (Fig. 4b–e). Compared to control rats, diabetic rats exhibited noticeably lower body weights and higher blood glucose levels, serum FFA levels, HbA1C levels, kidney weight/body weight ratios, and urine volumes. The kidney weight/body weight ratio and urine volume were not significantly altered in response to drug treatment. In addition, neither KF-1607 nor losartan affected the FFA levels or hyperglycemic status (Table 1).

**Table 1:** Characteristics of the experimental animals at the end of the study.

Group	Control	STZ	STZ + KF-1607	STZ + Losartan
Body weight (g)	509 ± 11	226 ± 8*	236 ± 9*	240 ± 8*
Kidney weight (g)	1.6 ± 0.1	1.4 ± 0.1	1.6 ± 0.1	1.4 ± 0.1
Kidney to body weight ratio (%)	0.3 ± 0.01	0.6 ± 0.01*	0.7 ± 0.03*	0.5 ± 0.03*
24-h urine volume (ml)	18 ± 0.4	103 ± 11.3*	94 ± 11.2*	84 ± 13.4*
HbA1C (%)	3.5 ± 0.1	7.0 ± 0.2*	6.9 ± 0.2*	7.2 ± 0.2*
Blood glucose (mg/dl)	153 ± 5	422 ± 39*	388 ± 17*	387 ± 3*
Serum FFA (mM)	0.24 ± 0.09	2.47 ± 0.78*	2.22 ± 0.49*	2.01 ± 0.28*

The data are presented as the mean ± SE of five rats/group.

*p < 0.05 vs. control.

The elevated serum creatinine and albuminuria suggested impaired kidney function in diabetic rats, and these phenomena were alleviated in KF-treated diabetic rats (Fig. 4f, g). Diabetic rats also showed an increased urinary level of KIM-1, a marker of tubular injury, and this level was significantly decreased in response to KF or losartan treatment (Fig. 4h). Kidney morphological changes, including an increased tuft area, glomerular volume, and FMA, were observed in kidney sections from diabetic rats and were attenuated following KF or losartan treatment (Fig. 4i–l).

### Pan-Src kinase inhibition alleviates ER stress and inhibits mTORC1 signaling in diabetic rat kidneys

**Fig. 5:**
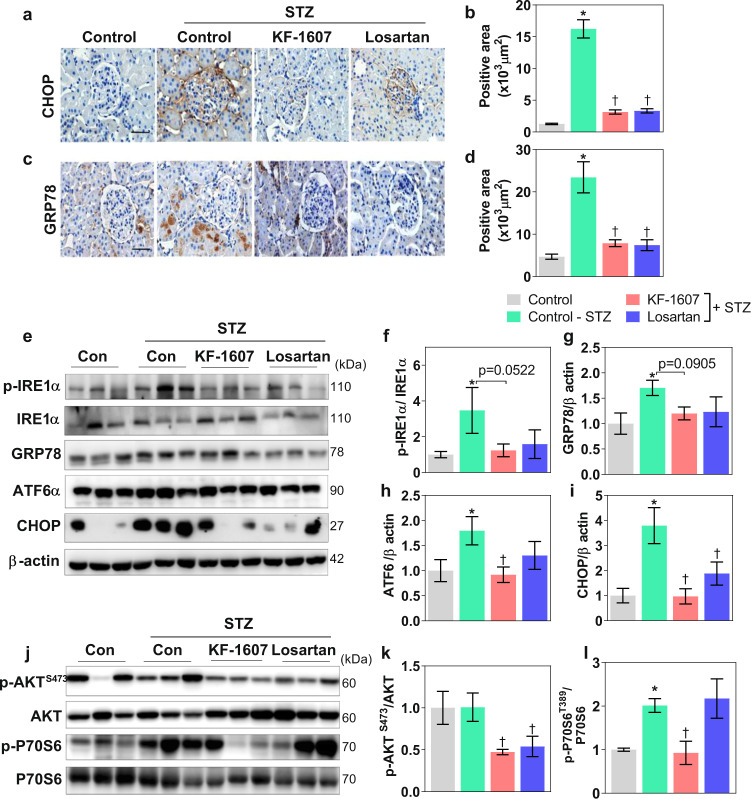
Src kinase inhibition downregulates ER stress and mTORC1 signaling in diabetic rats. Representative photomicrographs of kidney sections stained for **a** CHOP and **c** GRP78. The scale bar indicates 50 μm. **b**, **d** Quantitative analysis of the area of positive staining is presented. **e** ER stress signaling in the rat kidney was confirmed by immunoblot analysis of p-IRE1α, ATF6α, GRP78, and CHOP. **j** The protein levels of p-AKT^S473^ and p-p70S6K, which are associated with mTORC1 signaling, were determined. **f**–**i**, **k**, **l** The expression levels of the indicated proteins were quantified by densitometry. The data are presented as the mean ± SE of five rats. *p < 0.05 vs. control rats. ^†^*p* < 0.05 vs. control-STZ rats.

Exacerbation of ER stress was consistently shown in the kidneys of rats under diabetic stress, as indicated by the increased areas of positive CHOP and GRP78 staining in the tissue sections, which were decreased in both treatment groups (Fig. 5a–d). In addition, increased protein levels of phosphorylated IRE1α, GRP78, and ATF6α, as well as CHOP, were evident in diabetic kidneys, and these increases were attenuated in KF-treated rats. Losartan-treated rats showed a significant decrease in CHOP protein expression (Fig. 5e–i). In the present study, diabetes did not induce the phosphorylation of AKT^Ser473^, which is upstream of mTORC1. However, AKT^Ser473^ phosphorylation was significantly downregulated in both treatment groups. p70S6K phosphorylation was enhanced in diabetic kidneys and, interestingly, was decreased only in KF-treated rats (Fig. 5j–l).

### Inflammation, oxidative stress, and fibrosis in diabetic rat kidneys are attenuated in response to SKI treatment

**Fig. 6:**
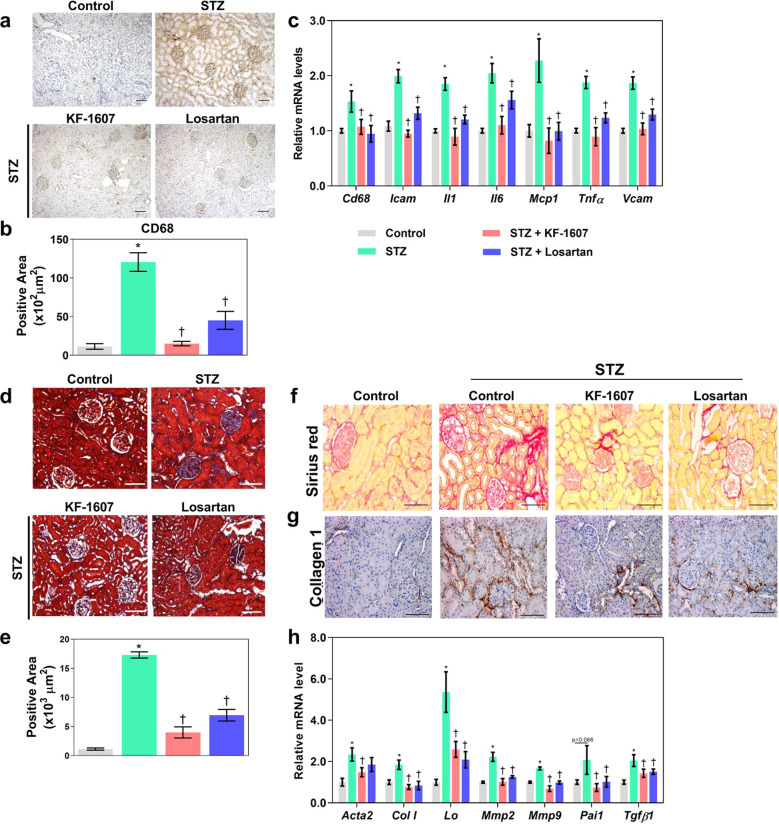
Src kinase inhibition ameliorates kidney inflammation and fibrosis in diabetic rats. **a** Representative photomicrographs and **b** quantitative analysis of CD68 staining in kidney sections. **c** The transcript levels of proinflammatory mediators were measured with RT–PCR. **d** Representative photomicrographs and **e** quantitative analysis of the fibrotic area as detected by Masson’s trichrome staining in kidney sections. The extracellular matrix was further confirmed with **f** picrosirus red and **g** collagen 1 IHC staining in kidney sections. The scale bar in all figures indicates 100 μm. **h** Fibrogenic gene expression was measured with RT–PCR. The data are presented as the mean ± SE of five rats. *p < 0.05 vs. control rats. ^†^*p* < 0.05 vs. control-STZ rats.

Following inhibition of ER stress signaling, we investigated whether pan-Src kinase inhibition might result in amelioration of inflammation and oxidative stress, which are excessively induced in kidneys under diabetic conditions^29^. Macrophage infiltration in diabetic kidneys, as detected by CD68 staining, was suppressed in response to drug treatment (Fig. 6a, b). Likewise, the mRNA levels of multiple proinflammatory mediators, i.e., *Cd68*, *Icam*, interleukin *(Il) 1β*, *Il6*, *Mcp1*, *Tnfα*, and *Vcam*, were reduced in both treatment groups (Fig. 6c). Furthermore, oxidative stress was enhanced in diabetic rats, as indicated by the increases in nitrotyrosine staining in kidney sections, plasma LPO, and urinary LPO. These increases were significantly reduced in KF- and losartan-treated rats (Supplementary Fig. 6a–d).

Moreover, we evaluated the preventive effect of SKI treatment against the development of kidney fibrosis, a common final outcome of kidney injury. Masson’s trichrome and picrosirius red staining showed increased collagen matrix accumulation in the diabetic kidneys, and this increase was significantly reduced by KF and losartan treatment (Fig. 6d–f). Collagen 1 protein accumulation in diabetic kidneys was also reduced in the treatment groups (Fig. 6g). Diabetic kidneys exhibited increased transcript levels of *Tgfβ1*, a major profibrotic regulator; lysyl oxidase (*Lo*), an enzyme that promotes the formation of the collagen fiber network in the ECM; *Acta2* and *Col 1*, mesenchymal-related markers; and *Mmp2* and *Mmp9*, inducers of epithelial–mesenchymal transition^30^. However, these levels were significantly decreased in both treatment groups (Fig. 6h). Collectively, our results showed the preventive effects of KF-1607 against inflammation, oxidative stress, and fibrosis and indicated that KF-1607 prevents these phenomena to the same extent as losartan.

### KF-1607 treatment shows protective effects on the rat tubular epithelium in the diabetic milieu

**Fig. 7:**
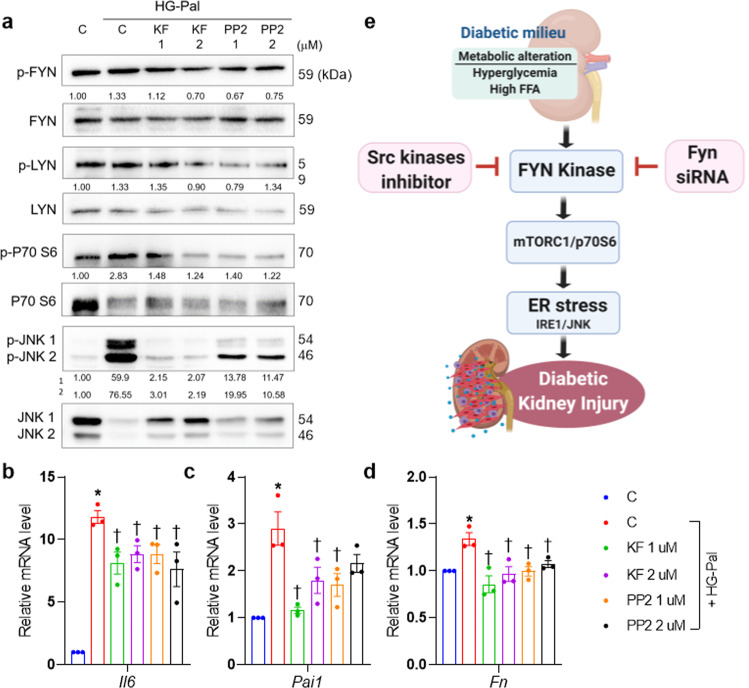
Protective effect of pan-SKI treatment on rat tubular epithelial cells in the diabetic milieu. NRK-52E cells were treated with KF-1607 in comparison with PP2 under insults mimicking diabetes. **a** The phosphorylation of Fyn and Lyn kinases, as well as p70S6K and JNK, was suppressed in response to pan-SKI treatment. **b**–**d** The transcript levels of genes related to inflammation and fibrosis were decreased in NRK-52E cells treated with pan-SKIs. The data are presented as the mean ± SE of three experiments. *p < 0.05 vs. Con, ^†^*p* < 0.05 vs. HG-Pal. **e** SFKs, particularly Fyn kinase, play a role in the development of DKD, partially through activation of ER stress signaling related to proximal tubular injury under diabetic conditions.

Given the comparable protective effects of KF-1607 and losartan on the kidneys in a diabetic murine model, we verified the direct protective effect of this novel compound in cultured rat kidney tubular epithelial cells (NRK-52E). Pretreatment of NRK-52E cells with KF-1607 exposed to HG-Pal inhibited Fyn and Lyn phosphorylation. Similar to the findings in the animal studies, pan-SKI treatment suppressed p70S6K and JNK activation (Fig. 7a). Furthermore, KF-1607 treatment markedly decreased the transcript levels of *Il6*, *Pai1*, and *Fn*, which are well-known inflammation and fibrosis markers (Fig. 7b–d).

## Discussion

Src kinase has been well identified to mediate the progression of kidney fibrosis, whereas the detailed mechanism by which Src kinase or other members of the SFKs mediate DKD has not been well elucidated. The present study demonstrated that Fyn and Lyn kinases were activated in diabetic rat kidneys and that treatment with a novel orally active pan-SKI suppressed ER stress and protected the kidneys against diabetic injury. Mechanistic studies with mouse proximal tubular cells further pinpointed the role of Fyn kinase, a specific member of the SFKs, as a positive regulator of ER stress and tubular injury under diabetic stress.

Fyn kinase has been identified as a member of the SFKs that is particularly involved in the regulation of metabolic functions, including insulin sensitivity and adipose tissue homeostasis, in vivo^31,32^. Here, we demonstrated that high glucose–palmitate exposure induced activation of Fyn and Lyn kinases, increased ER stress signaling, and activated the p70S6K protein in mouse proximal tubular cells. *Fyn*-specific siRNA transfection decreased the levels of phosphorylated p70S6K (a major downstream target of mTORC1), phosphorylated JNK (a downstream target of IRE1α), and CHOP (a transcription factor responsible for ER stress-related apoptosis) in HG-Pal-treated mProx24 cells. mTORC1 has been reported to act upstream of IRE1-JNK signaling. Rapamycin, an mTORC1 inhibitor, was found to suppress IRE1-JNK signaling in NRK-52E rat renal tubular epithelial cells^33^. Furthermore, Fyn overexpression in skeletal muscle was shown to stimulate mTORC1, leading to IRE1α-JNK activation and apoptosis induction^13^.

Src kinase was shown to induce mTORC1 recruitment and activation at the lysosomal surface by promoting the dissociation of the Rag-GATOR1 complex through an unclear mechanism^28^. It has also been reported that palmitate activates mTORC1 signaling by enhancing mTORC1 translocation to the lysosomal membrane, resulting in ER stress activation and apoptosis in podocytes^26^. In the present study, mTOR translocation to the lysosomal membrane was enhanced under HG-Pal stimulation but was inhibited by Fyn knockdown. This pattern implies that Fyn might induce the translocation of mTOR to the lysosomal membrane in tubular cells in the diabetic milieu.

In addition, Fyn overexpression has been shown to inhibit the LKB1-AMPK pathway, which results in mTORC1 activation^34,35^. Our in vitro study showed elevated AMPKα protein expression under HG-Pal stimulation, and this increase was enhanced under SKI treatment and Fyn knockdown. The gene expression levels of Ampkα1 and Ampkα2 and the protein level of phosphorylated ULK1^Ser317^, a direct downstream target of AMPK, were also increased in Fyn knockdown cells under HG-Pal stimulation (Supplementary Fig. 7). Decreased AMPK activity has generally been demonstrated to occur under diabetic conditions^36,37^. Surprisingly, AMPK activity was not decreased in our STZ-induced diabetic rats. Moreover, we did not observe any changes in AMPK signaling in response to KF compound treatment (data not shown).

Pharmacological inhibition of pan-Src kinases has been demonstrated to markedly inhibit tubular cell apoptosis in the diabetic milieu^12^. The present study showed that genetic ablation of *Fyn* but not *Lyn* decreased p70S6K and JNK-CHOP expression as well as tubular cell apoptosis in HG-Pal-stimulated mProx24 cells. In support of our finding, IgE/antigen-induced bone marrow-derived mast cells treated with *Fyn* siRNA but not *Lyn* siRNA showed downregulation of p70S6K and JNK phosphorylation^38^. Interestingly, a study reported the opposite effects of Lyn and Fyn kinases in a nephritis model, where Fyn deficiency was associated with protection against nephritis^39^. Regardless of the unclear mechanism and significance of the Fyn–Lyn balance in the diabetic kidney, the present study mainly highlighted the important role of Fyn kinase, a specific member of the SFKs, in inducing ER stress-mediated tubular apoptosis under diabetic stress.

In agreement with the in vitro results, the levels of phosphorylated Fyn and Lyn kinases were increased in the kidneys of diabetic rats. Pan-Src kinase inhibition decreased the phosphorylation of these SFK members and reduced ER stress and mTORC1 activity in diabetic kidneys. The present data highlighted the involvement of other SFKs in addition to Src kinase in the pathogenesis of DKD. In line with our results, database analysis showed that the transcript levels of Fyn and Lyn kinases but not Src kinase were elevated in STZ-DBA/2J mice and db/db mice^24^ as well as in renal tubules in human kidney biopsy samples from the *Woroniecka* dataset^22^. Our results were quite different from those of other studies, which primarily showed activation of Src kinase in diabetic db/db mice^12^, STZ-DBA2/J mice^6^, and STZ-induced SD rats during modeling for 24 weeks^40^. This discrepancy might be related to the different animal strains and durations of diabetes used in the in vivo studies.

Treatment with the pan-SKI KF-1607 significantly prevented inflammation, oxidative stress, and fibrosis and attenuated structural and functional changes in the diabetic kidney without affecting the systemic blood glucose level. KF-1607 treatment attenuated kidney dysfunction and tubular injury, as shown by the decreases in the serum creatinine level, albuminuria, and the urinary KIM-1 level. The renoprotective effect of KF-1607 was consistent with other results in STZ-induced diabetic mice^6^ and db/db mice^12^ treated with PP1, an experimental pan-SKI. However, a further study with delayed KF-1607 treatment remains necessary to verify its therapeutic effects in DKD.

In addition, we demonstrated that the renoprotective effect of KF-1607 was similar to that of losartan. Similar to our present results, losartan has been shown to ameliorate ER stress-induced aggravation of kidney injury, such as that occurring in diabetic OVE26 mice^41^ and in apoE knockout mice with 5/6 nephrectomy^42^. In our in vitro study with mProx24 cells, losartan treatment attenuated mTORC1 and ER stress activation under HG-Pal exposure (Supplementary Fig. 8). The renin–angiotensin system has been identified as an upstream regulator of mTORC1 activity^43^, and a study with Ren2 hypertensive rats reported decreases in phosphorylated mTORC1 and p70S6K levels in the kidney following telmisartan treatment^44^. However, suppression of mTORC1/p70S6K activation was not observed in losartan-treated diabetic rats in our present study. This discrepancy is likely due to differences in the treatment duration and dose of ARB that might contribute to its efficacy in modulating the mTORC1 signaling pathway.

A few studies have elucidated the effects of Src kinase inhibition on RAAS activity^45,46^. In the present study, the gene expression levels of RAAS components were increased in the diabetic kidneys of our experimental rats. The relative mRNA levels of angiotensinogen, ACE, and AT1R tended to decrease in KF-treated diabetic rats (Supplementary Fig. 9). However, our results are too preliminary to delineate the relationship between SFKs and the RAAS in the diabetic kidney. Since the RAAS is a tightly regulated system and SFKs can be activated not only by Ang II but also by other factors activated during diabetes, further studies are necessary to understand the exact effect of SKIs on RAAS signaling in diabetic kidneys. It also remains interesting to investigate the effects of combined treatment with KF-1607 and losartan.

Previous studies have supported the idea that pan-SKIs targeting multiple pathways might significantly inhibit kidney inflammation and fibrosis, which are mediated by diverse key pathological events and signaling activation^9,11^. On the other hand, the homology and broad expression of SFK members suggest that the safety of pan-SKIs is an important consideration for their clinical use^47^. The hERG automatic patch clamp assay indicated the low cardiotoxic potency of KF-1607. This new scaffold also exhibited lower toxicity than its precursor, ponatinib, in an in vitro kinetic cytotoxicity assay with renal mesangial cells^10^. Moreover, a recent phase IIa clinical study with daily use of saracatinib suggested that a safe and tolerable small molecule inhibitor could be used for chronic therapy of a nonmalignant disease^48^. However, studying the mechanistic role of a specific member of the SFKs with the available inhibitors, which target multiple SFK members, remains challenging. Although our in vitro data with siRNA-based gene knockdown demonstrated the involvement of Fyn in ER stress-mediated DKD, future studies with kidney-specific Fyn knockout mice under DKD induction should be conducted to strengthen the current evidence.

In summary, the present study demonstrated that Fyn and Lyn kinase expression was induced in response to diabetic kidney injury. *Fyn* knockdown but not *Lyn* knockdown inhibited P70S6K and JNK/CHOP signaling, resulting in suppression of proximal tubular cell injury in the diabetic milieu. Treatment with a pan-SKI attenuated ER stress signaling and ameliorated inflammation, oxidative stress, and structural and functional evidence of progressive DKD, with trends similar to those observed in rats treated with losartan (Fig. 7e). Thus, our present study provides validation for Fyn kinase as a viable target for the development of a therapeutic agent for DKD.

## Supplementary information

Supplemental Material
