# Supplementary material for: Pan-Src kinase inhibitor treatment attenuates diabetic kidney injury via inhibition of Fyn kinase-mediated endoplasmic reticulum stress
Source: Exp Mol Med. 2022 Aug 2;54(8):1086–97. doi: 10.1038/s12276-022-00810-3 (PMC9440146; doi:10.1038/s12276-022-00810-3)
Supplement: Supplementary file 1 — Supplemental Material [file 12276_2022_810_MOESM1_ESM.docx]

**SUPPLEMENTARY MATERIALS**

**Pan-Src kinase inhibitor treatment attenuates diabetic kidney injury via inhibition of Fyn kinase-mediated endoplasmic reticulum stress**

Debra Dorotea^1^, Songling Jiang^1^, Eun Seon Pak^1^, Jung Beom Son^2^, Hwan Geun Choi^2^, Sung-Min Ahn^3^, and and Hunjoo Ha^1*^


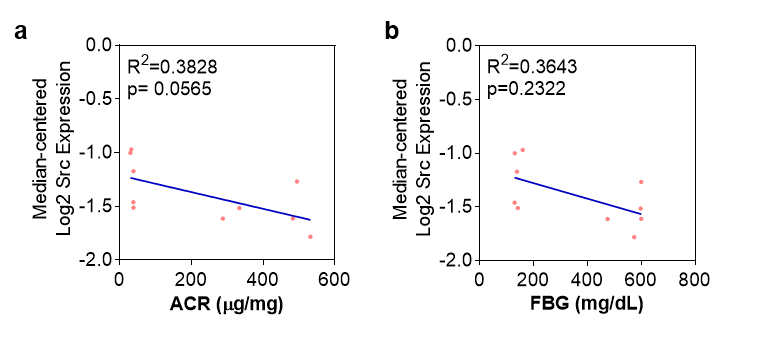


**Supplementary Fig. 1. Src kinase is not corellated to diabetic kidney disease.** The corelation of *Src* transcript level with **a** albumin/creatinine ratio (ACR) and **b** plasma fasting blood glucose (FBG) from *Hodgin* dataset ([GSE33744](https://www.ncbi.nlm.nih.gov/geo/query/acc.cgi?acc=GSE33744)) that includes control and db/db mice.


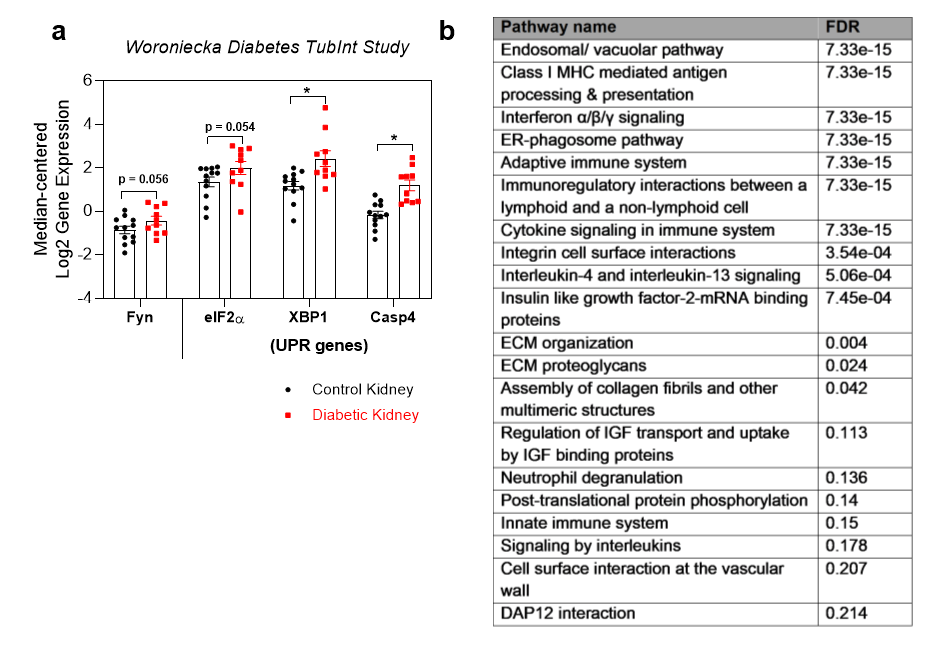


**Supplementary Fig. 2. Biochemical pathways enriched in human diabetic kidney.** Dataset analysis of Woroniecka Tubulointl Diabetes Study (GSE30122). **a** mRNA expression of *FYN* and unfolded protein response (UPR)-related genes. **b** Statistically significant enrichment of *Reactome* pathways for differentially expressed genes in the diabetic tubules. FDR *p*-value < 0.05.


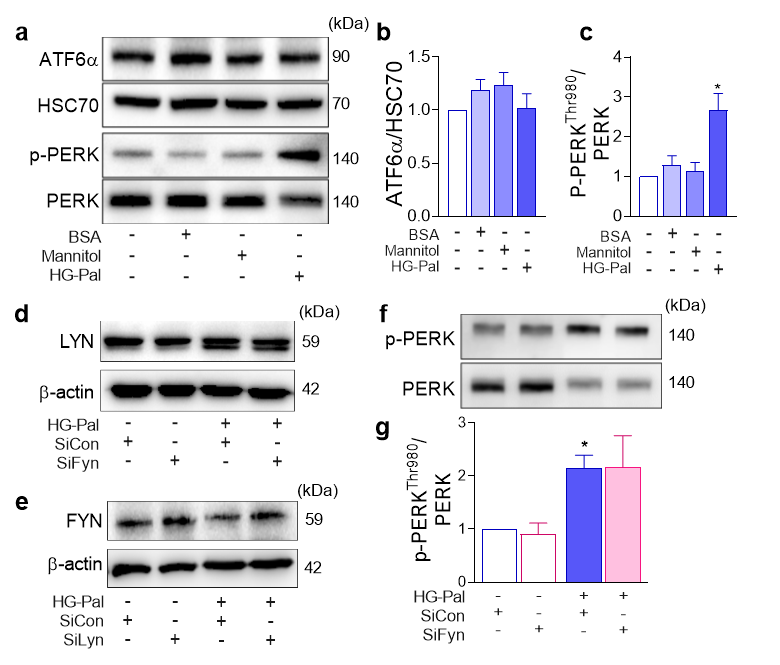


**Supplementary Fig. 3.** **Knockdown of *Fyn* does not suppress phosphorylated PERK in HG-Pal-induced mProx24 cells.** mProx24 cells were treated with HG-Pal for 10 h. Cell lysates were subject to immunoblot analysis. **a** Representative immunoblots of ATF6α and phosphorylated PERK. **d** The specificity of *Fyn* and **e** *Lyn* siRNA was examined. **f** Representative immunoblots of phospho- and total- PERK in mProx24 cells transfected with scrambled siRNA or *Fyn* siRNA with and/or without HG-Pal stimulation. **b,c,g** Expression levels of indicated protein were quantified with densitometry. Data are represented as means ± SE of 4 experiments. ^*^*p*<0.05 vs. control.


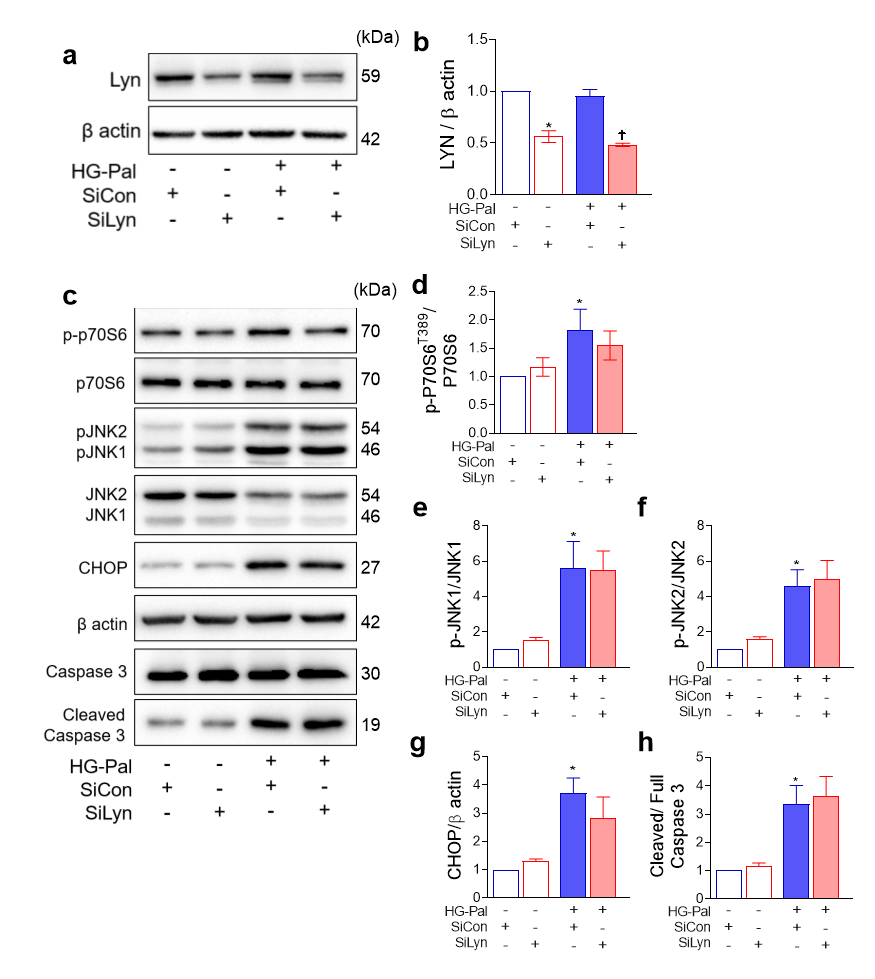


**Supplementary Fig. 4.** **Knockdown of *Lyn* does not affect HG-Pal-induced mTORC1/ER stress activation in mProx24 cells.** mProx24 cells were transfected with scrambled siRNA or *Lyn* siRNA with and/or without HG-Pal stimulation. Cell lysates were subject to immunoblot analysis. **a** Representative immunoblots and **b** quantitative analyses of Lyn protein expression. **c** Representative immunoblots of phosphorylated P70S6 kinase (P70S6^T389^), P70S6 kinase, phosphorylated JNK1/2^T183, Y185^, JNK 1/2, CHOP, caspase 3, and cleaved caspase 3. **d-h** Expression levels of indicated protein were quantified with densitometry. Data are represented as means ± SE of 4 experiments. ^*^*p*<0.05 vs. siCon, ^†^ *p*<0.05 vs. HG-Pal and siCon.


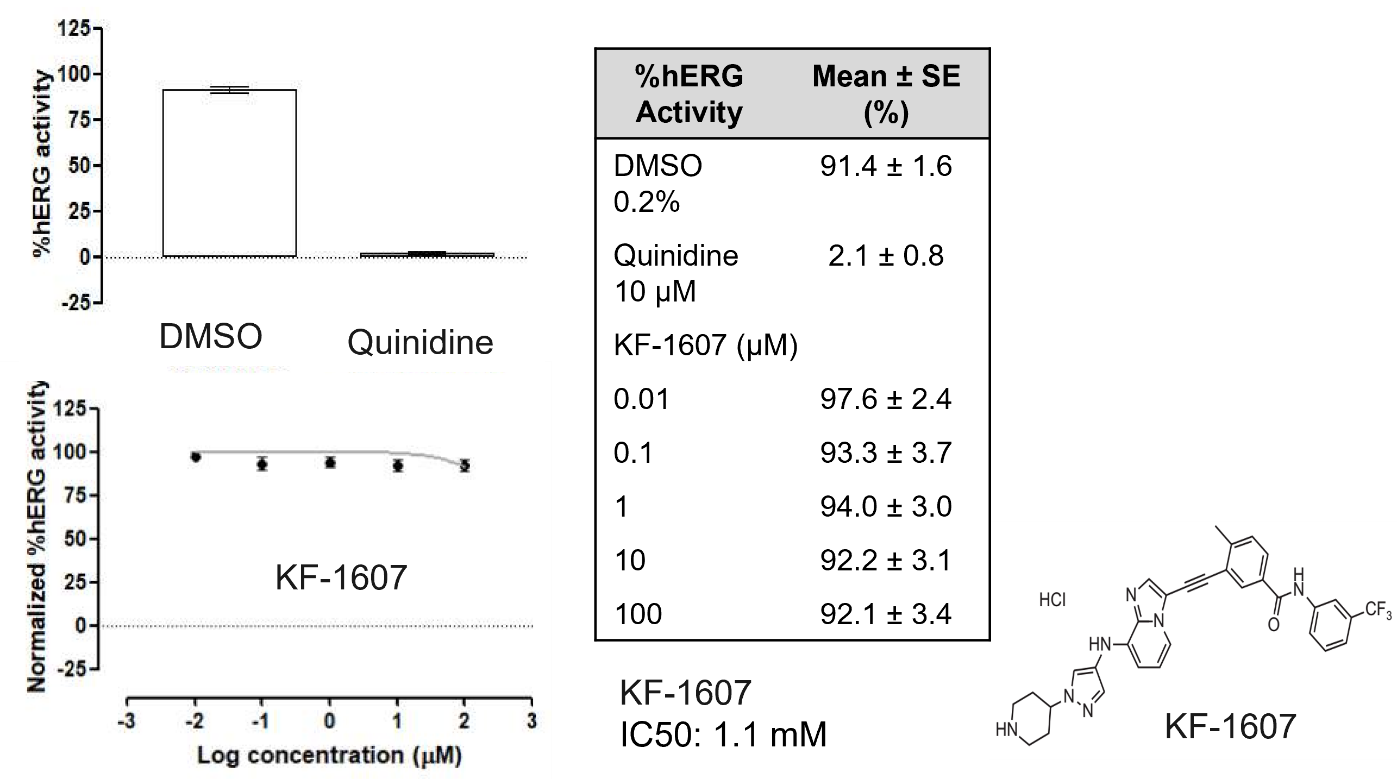


**Supplementary Fig. 5. The potential cardiotoxicity of KF-1607.** hERG automated patch clamping assay was performed to verify the potential cardiotoxicity of KF-1607 (4-methyl-3-((8-((1-(piperidin-4-yl)-1H-pyrazol-4-yl)amino)imidazo[1,2-a]pyridin-3-yl)ethynyl)-N-(3-(trifluoromethyl)phenyl) benza mide hydrochloride salt).


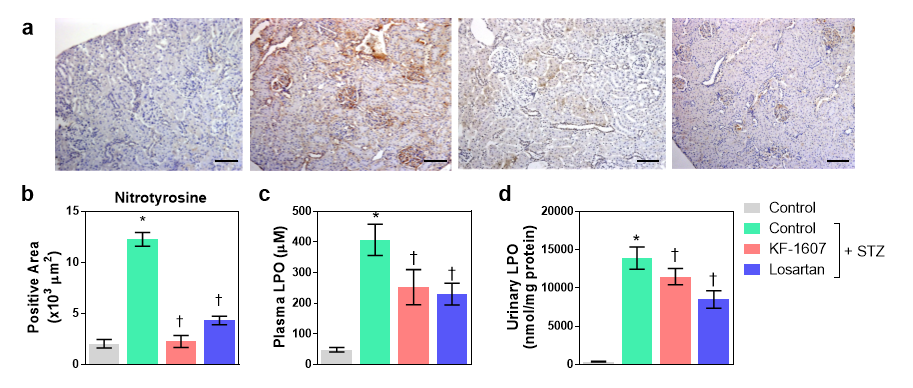


**Supplementary Fig. 6.** **Src kinase inhibition decreases kidney oxidative stress in diabetic rats.** **a** Representative photomicrographs of nitrotyrosine-stained kidney sections. The scale bar indicates 100 μm. **b** Quantitative analyses of positive staining area are depicted. Lipid peroxide (LPO) levels in the **c** plasma and **d** urine of experimental rats were measured. Values are expressed as means ± SE of 5 rats. **p*<0.05 vs. control rats. ^†^*p*<0.05 vs. control-STZ rats.

**
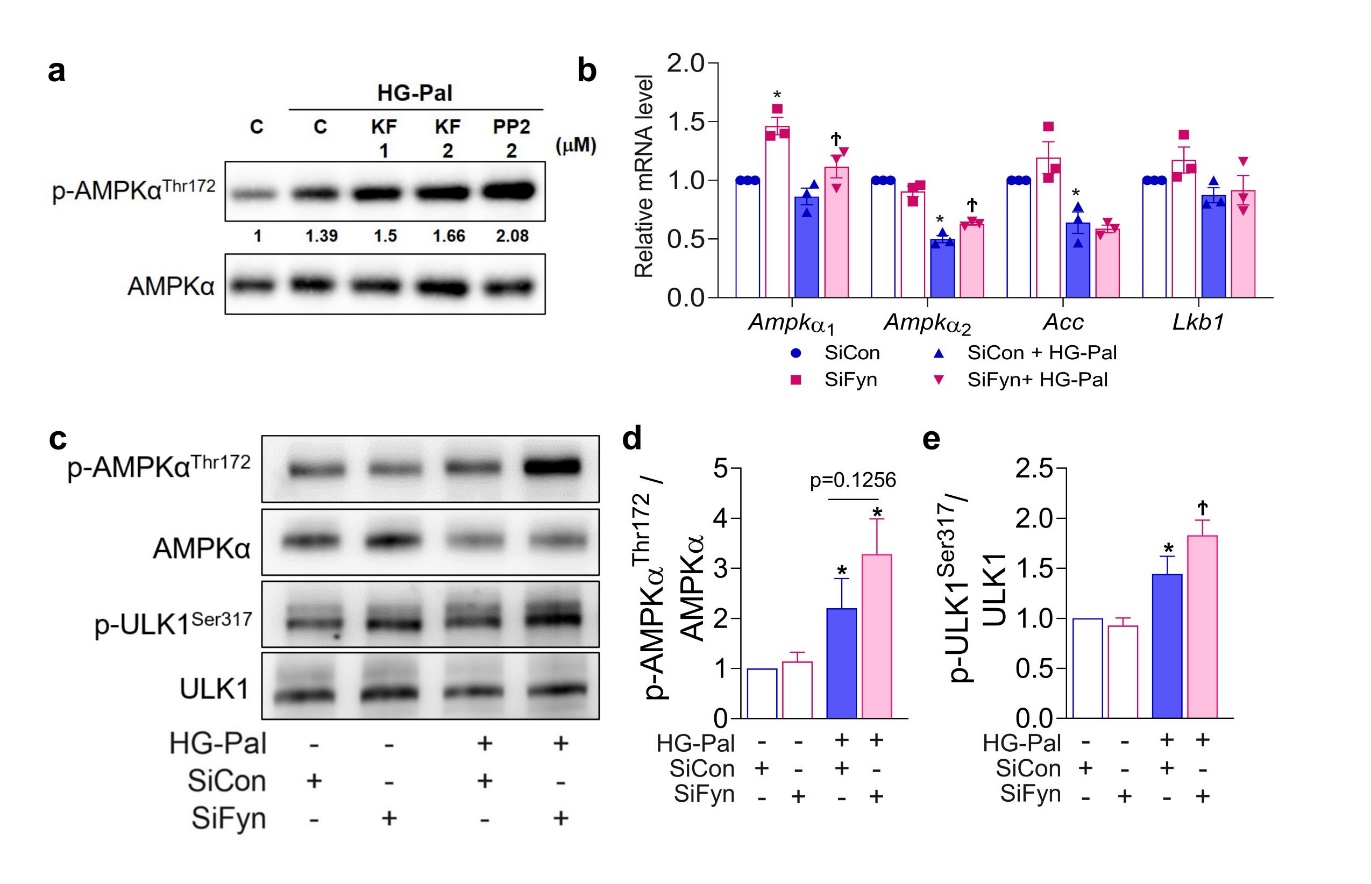
**

**Supplementary Fig. 7.** **Src kinase inhibitor and knockdown of Fyn kinase modulate AMPK expression in HG-Pal-induced mProx24 cells. a** mProx24 cells were treated with KF-1607, in comparison with PP2, under HG-Pal conditions. Protein expressions of phosphorylated AMPKα^T172^ and AMPKα were determined. **b** mProx24 cells were transfected with scrambled siRNA or Fyn siRNA with and/or without HG-Pal stimulation. Relative mRNA levels of Ampkα1, Ampkα2, Lkb1, and Acc. **c** Cell lysates were subject to immunoblot analysis. Representative immunoblots of phosphorylated AMPKα^T172^, AMPKα, phosphorylated ULK1^S317^, and ULK1. **d, e** Expression levels of indicated protein were quantified with densitometry. Data are represented as the mean ± SE of 4 experiments. ^*^*p*<0.05 vs. siCon, ^†^ *p*<0.05 vs. HG-Pal and siCon.

**
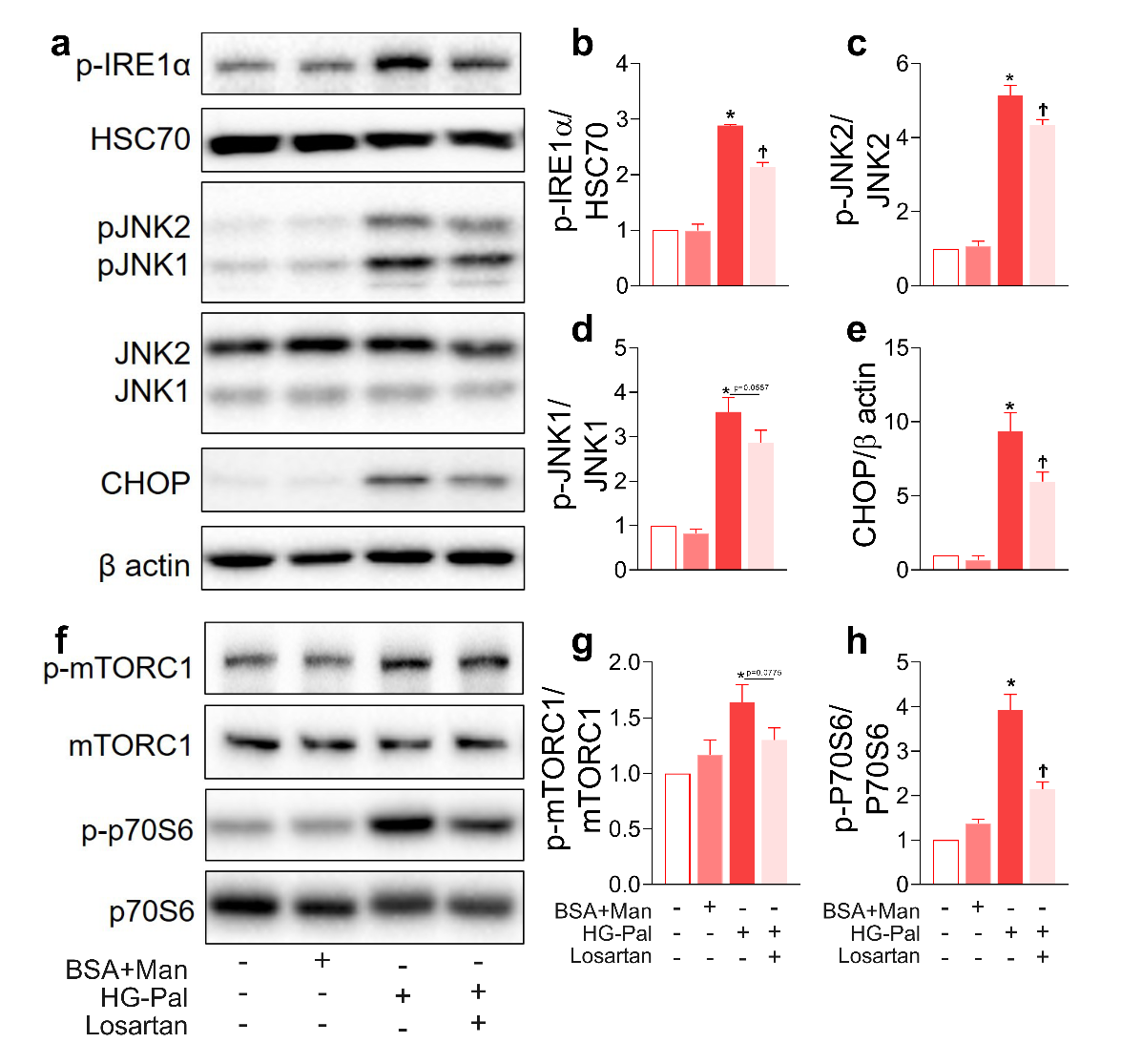
**

**Supplementary Fig. 8. Losartan suppresses HG-Pal-induced ER stress/ mTORC1 activation in mProx24 cells.** mProx24 cells were treated with losartan 1 μM one hour prior to stimulation with HG-Pal. Cell lysates were subject to immunoblot analysis. **a,f** Representative immunoblots of phosphorylated IRE1α ^S724^, phosphorylated JNK1/2^T183, Y185^, JNK 1/2, CHOP, phosphorylated mTORC1^S2448^, mTORC1, phosphorylated P70S6 kinase (P70S6^T389^), and P70S6 kinase. **b-e, g, h** Expression levels of indicated protein were quantified with densitometry. Data are represented as means ± SE of 3 experiments. ^*^*p*<0.05 vs. Con, ^†^ *p*<0.05 vs. HG-Pal.


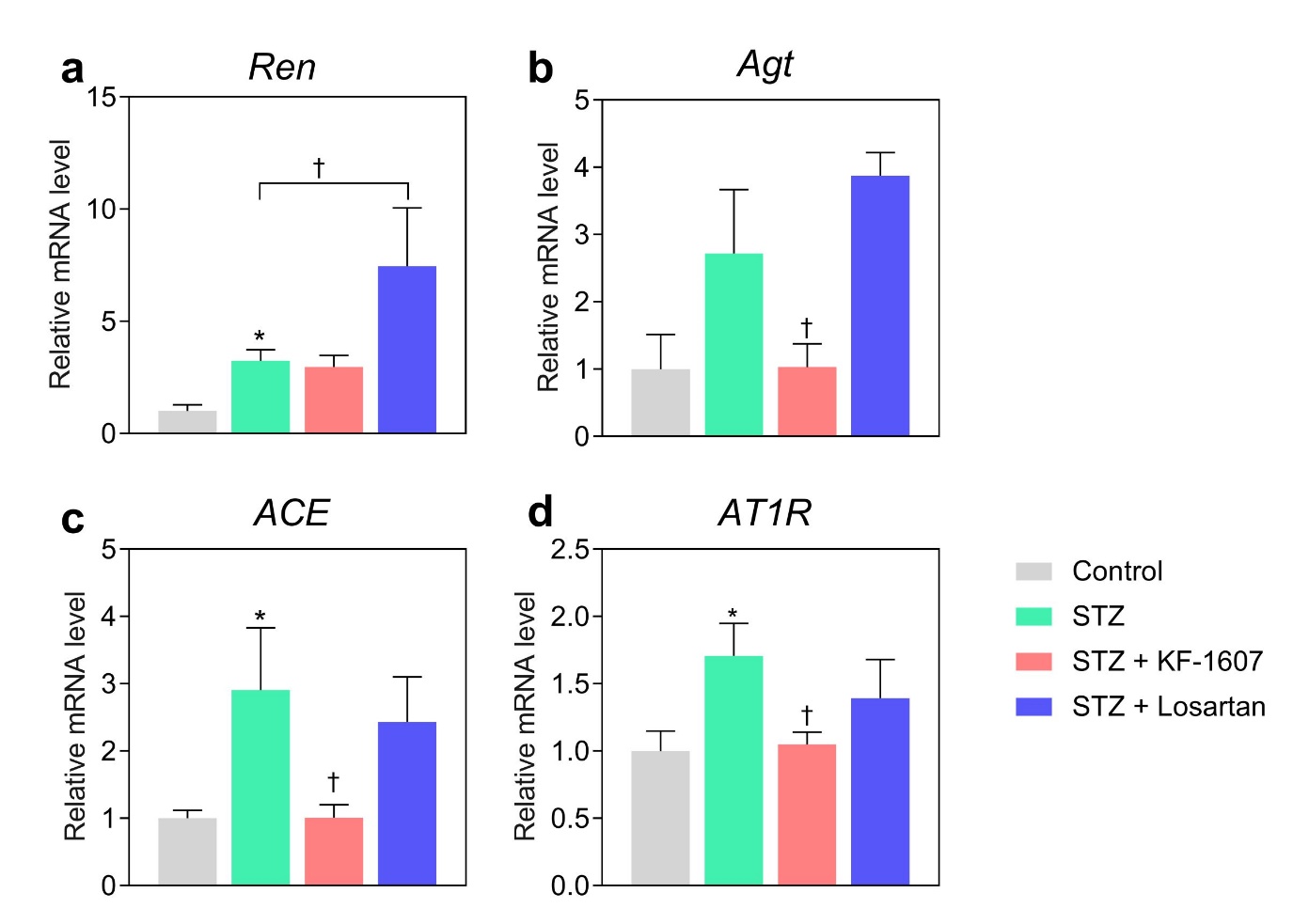


**Supplemenary Fig. 9. The effect of Src kinase inhibition on renin-angiotensin system in the kidney.** Relative mRNA levels of **a** renin (Ren), **b** angiotensinogen (Agt), **c** angiotensin converting enzyme (ACE), and **d** angiotensinogen II type 1 receptor (AT1R). **p*<0.05 vs. control rats. ^†^*p*<0.05 vs. control-STZ rats.

**Supplementary Table 1. Primary antibodies used in the present study**

| **Antibody** | **Use & dilution** | **Cat. number** | **Source** |
| --- | --- | --- | --- |
| anti-AKT | WB 1:1000 | #9272 | Cell Signaling, Danvers, MA, USA |
| anti-AMPKα | WB 1:1000 | #2532 | Cell Signaling |
| anti-ATF6α | WB 1:1000 | sc166659 | Santa Cruz Biotechnology, CA, USA |
| anti-Caspase 3 | WB 1:1000 | #9662 | Cell Signaling |
| anti-CD68 | IHC 1:400 | sc13572 | Santa Cruz |
| anti-CHOP  (GADD 153) | WB 1:500; IHC 1:400; IF 1:200 | ab11419 | Abcam, Waltham, MA, USA |
| anti-Cleaved caspase 3 | WB 1:1000 | #9661 | Cell Signaling |
| anti-Collagen 1 | IHC 1:400 | 1310-01 | Southern Biotech, Birmingham, AL, USA |
| anti-Fyn | WB 1:400 | sc365913 | Santa Cruz |
| anti-GRP78 | WB 1:500; IHC 1:400 | sc166490 | Santa Cruz |
| anti-HSC70 | WB 1:2000 | sc7298 | Santa Cruz |
| anti-JNK | WB 1:1000 | #9252 | Cell Signaling |
| anti-LAMP1 | IF 1:500 | sc20011 | Santa Cruz |
| anti-LYN | WB 1:1000 | #2796 | Cell Signaling |
| anti-mTOR | IF 1:500 | #2972 | Cell Signaling |
| anti-Nitrotyrosine | IHC 1:400 | sc32757 | Santa Cruz |
| anti-P70SK | WB 1:1000 | #2708 | Cell Signaling |
| anti-p-AKT^Ser473^ | WB 1:1000 | #9271 | Cell Signaling |
| anti-p-AMPKα^Thr172^ | WB 1:1000 | #2531 | Cell Signaling |
| anti-PERK | WB 1:1000 | #3192 | Cell Signaling |
| anti-p-FYN | WB 1:500; IF 1:200 | sc377555 | Santa Cruz |
| anti-p-IRE1α^Ser724^ | WB 1:1000; IF 1:200 | NB100-2323 | Novus Biologicals, Centennial, CO, USA |
| anti-p-JNK^Thr183, Tyr185^ | WB 1:1000 | #9251 | Cell Signaling |
| anti-p-LYN | WB 1:1000; IF 1:200 | bs3251 | Bioss, Woburn, MA, USA |
| anti-p-P70S6K | WB 1:1000 | #9234 | Cell Signaling |
| anti-p-PERK | WB 1:1000 | #3179 | Cell Signaling |
| anti-p-ULK1^Ser317^ | WB 1:1000 | #12753 | Cell Signaling |
| anti-ULK1 | WB 1:1000 | A7481 | Sigma-Aldrich |
| anti-β actin | WB 1:2000 | A5411 | Sigma-Aldrich |

*IHC immunohistochemistry, IF immunofluorescence, WB western blot.

**Supplementary Table 2. Primer sequences used in the present study**

| **Gene** | **Forward primer (5’-3’)** | **Reverse primer (5’-3’)** |
| --- | --- | --- |
| **Mouse** |  |  |
| *18S* | CGA AAGCAT TTGCCAAGA AT | AGT CGGCATCGTTTATGG TC |
| *Acc* | GTCCGCACTGACTGTAACCA | GCCAGACTCGTTTGTCAGGA |
| *Ampkα1* | AACGCATTTGGAGGACATGA | TTGTCCGGAAATCAGTGCAT |
| *Ampkα2* | AAAGGATGCCGCCTCTCATC | GGCTTCGTTGTGTTGAGTGC |
| *Fyn* | CTTTGGGGGTGTGAACTCCT | TTCTGCCTGGATGGAGTCAA |
| *Il6* | AGTTGCCTTCTTGGGACTGA | TCCACGATTTCCCAGAGAAC |
| *Kim1* | ACATATCGTGGAATCACAACGAC | ACAAGCAGAAGATGGGCATTG |
| *Lkb1* | CTACTCCGAGGGATGTTGGA | GATAGGTACGAGCGCCTCAG |
| *Lyn* | AGCTCCAGAGGCCATCAACT | CACATCTGCGTTGGTTCTCC |
| *Mcp1* | CTTCTGGGCCTGCTGTTCA | CCAGCCTACTCATTGGGATCA |
| **Rats** |  |  |
| *18s* | AGGAATTGACGGAAGGGCAC | GTGCAGCCCCGGACATCTAAG |
| *ACE* | AGGTTCGTGGAGGAGTATGA | TTGCTGCCCTCTATGGTAATG |
| *Acta2* | GATCACCATCGGGAATGAACGC | CTTAGAAGCATTTGCGGTGGA |
| *Agt* | CACGGACAGCACCCTATTT | GTTGTCCACCCAGAACTCAT |
| *AT1R* | GCGCTCATCAGACTGTAGATAAT | CAGCTCCTGACTCTTCCTTTG |
| *Cd68* | CGTTACCCGGAGACGACAAT | TCCTTGGTGGCCTACAGAGT |
| *Col 1* | GGTTCACCACTGTTGCCTTT | AATGGTGCTCCTGGTATTGC |
| *Fn* | ACAACAGGAGAGTAGGGCGC | TGTGACCAGCAACACGGTG |
| *Icam* | TGCACGTCCCTGGTGATACTC | AAACGGGAGATGAATGGTACCTAC |
| *Il1* | GACTTCACCATGGAACCCGT | GGAGACTGCCCATTCTCGAC |
| *Il6* | TCTCTCCGCAAGAGACTTCCA | ATACTGGTCTGTTGTGGGTGG |
| *Lo* | GTAGCAGTACCCTGTGGTCATAGTC | TCCACGTACGTACAAAAGATGTCTA |
| *Mcp1* | CTGTAGCATCCACGTGCTGT | CCGACTCATTGGGATCATCT |
| *Mmp 2* | CTTTGTGCTGAAAGATACCC | AAGTTGTAGTTGGCCACATC |
| *Mmp 9* | CCACCGAGCTATCCACTCAT | GTCCGGTTTCAGCATGTTTT |
| *Pai1* | TCCGCCATCACCAACATTTT | GTCAGTCATGCCCAGCTTCTC |
| *Ren* | CTATGACTCCTCGGAATCCTCTA | CACCCACAGTTACCACATCTT |
| *Tgfβ1* | GCTCCACAGTTGACTTGAAT | TGACAAAACCAAAGACATCA |
| *Tnfα* | CAGCAGATGGGCTGTACCTT | AAATGGCAAATCGGCTGACG |
| *Vcam* | TGCACGGTCCCTAATGTGTA | TGCCAATTTCCTCCCTTAAA |
